# Long non-coding RNA ANRIL is upregulated in hepatocellular carcinoma and regulates cell apoptosis by epigenetic silencing of KLF2

**DOI:** 10.1186/s13045-015-0146-0

**Published:** 2015-05-14

**Authors:** Ming-de Huang, Wen-ming Chen, Fu-zhen Qi, Rui Xia, Ming Sun, Tong-peng Xu, Li Yin, Er-bao Zhang, Wei De, Yong-qian Shu

**Affiliations:** 1 Department of Medical Oncology, Huai’an First People’s Hospital, Nanjing Medical University, Huai’an City, Jiangsu Province 223301 China; 2 Department of Oncology, Jining No. 1 People’s Hospital, No. 6, Jiankang Road, Jining City, Shandong Province 272011 China; 3 Department of Hepatopancreatobiliary Surgery, Huai’an First People’s Hospital, Nanjing Medical University, Huai’an City, Jiangsu Province 223300 China; 4 Department of Biochemistry and Molecular Biology, Nanjing Medical University, Nanjing City, Jiangsu Province China; 5 Department of Oncology, First Affiliated Hospital, Nanjing Medical University, Nanjing City, Jiangsu Province China

**Keywords:** Long non-coding RNA, ANRIL, HCC, Proliferation, KLF2

## Abstract

**Background:**

Hepatocellular carcinoma (HCC) is one of the leading causes of cancer-related death, especially in China. And the mechanism of its progression remains poorly understood. Growing evidence indicates that long non-coding RNAs (lncRNAs) are found to be dysregulated in many cancers, including HCC. ANRIL, a lncRNA co-clustered mainly with *p14/ARF* has been reported to be dysregulated in gastric cancer, esophageal squamous cell carcinoma, and lung cancer. However, its clinical significance and potential role in HCC are still not documented.

**Methods and results:**

In this study, expression of ANRIL was analyzed in 77 HCC tissues and matched normal tissues by using quantitative polymerase chain reaction (qRT-PCR). ANRIL expression was upregulated in HCC tissues, and the higher expression of ANRIL was significantly correlated with tumor size and Barcelona Clinic Liver Cancer (BCLC) stage. Moreover, taking advantage of loss-of-function experiments in HCC cells, we found that knockdown of ANRIL expression could impair cell proliferation and invasion and induce cell apoptosis both *in vitro* and *in vivo*. We also found that ANRIL could epigenetically repress Kruppel-like factor 2 (KLF2) transcription in HCC cells by binding with PRC2 and recruiting it to the KLF2 promoter region. We also found that SP1 could regulate the expression of ANRIL.

**Conclusion:**

Our results suggest that lncRNA ANRIL, as a growth regulator, may serve as a new biomarker and target for therapy in HCC.

**Electronic supplementary material:**

The online version of this article (doi:10.1186/s13045-015-0146-0) contains supplementary material, which is available to authorized users.

## Background

Hepatocellular carcinoma (HCC) is the third leading cause of cancer-related death globally. Half of these deaths were estimated to occur in China [1]. The prognosis of patients with HCC remains poor despite the therapeutic advances in HCC treatment recently. Therefore, a great challenge lies ahead in the understanding of the molecular mechanisms of hepatocarcinogenesis and the identification of the new biomarkers for HCC that will supply an arm for improving diagnosis and management of human HCC.

lncRNAs are non-protein-coding transcripts with a length greater than 200 nucleotides. Accumulating evidence showed that lncRNAs participated in cancer cell biological processes, such as cell growth, cell metastasis, cell differentiation, and fate decision [2-4]. Additionally, many studies demonstrate that lncRNAs play a critical role in tumorigenesis, and their misexpression confers tumor initiation, cancer cell growth, and metastasis [5-7]. For example, lncRNA HOTAIR is dysregulated in many cancers [8,9]. Moreover, it could promote the invasion-metastasis cascade in cancer cells by binding to PRC2 [8]. In a word, there has been a heavy focus on the ways that lncRNAs contribute to cancer development. However, their aberrant expression and functional roles in HCC development are still not well documented.

Among them, lncRNA ANRIL (CDKN2B antisense RNA 1) is transcribed from the INK4b-ARF-INK4a gene cluster in the opposite direction, which has been identified as a shared genetic susceptibility locus associated with coronary disease, intracranial aneurysm, type 2 diabetes, and also cancers [10,11]. Moreover, ANRIL could be induced by the ATM-E2F1 signaling pathway and is required for the silencing of p15INK4B by recruiting PRC2 [12,13]. In our previous study, we found that ANRIL was overexpressed and played an important role in gastric carcinogenesis and NSCLC development [14,15]. However, the functional role and underlying mechanism of ANRIL in HCC remain unclear. Here we investigate the relationship between ANRIL and HCC. We found that ANRIL was upregulated in HCC tissues than in corresponding non-tumor tissues and its upregulation is related with tumor size and Barcelona Clinic Liver Cancer (BCLC) stage. Moreover, ANRIL could regulate cell growth both *in vitro* and *in vivo* via epigenetic silencing of Kruppel-like factor 2 (KLF2) by binding to PRC2. We also found that SP1 could regulate the expression of ANRIL. Our results suggest that SP1-induced ANRIL can regulate KLF2 expression in the epigenetic level and facilitate the development of lncRNA-directed diagnostics and therapeutics of HCC.

## Results

### ANRIL is upregulated in hepatocellular carcinoma tissues and is associated with tumor size and BCLC stage

**Figure 1:**
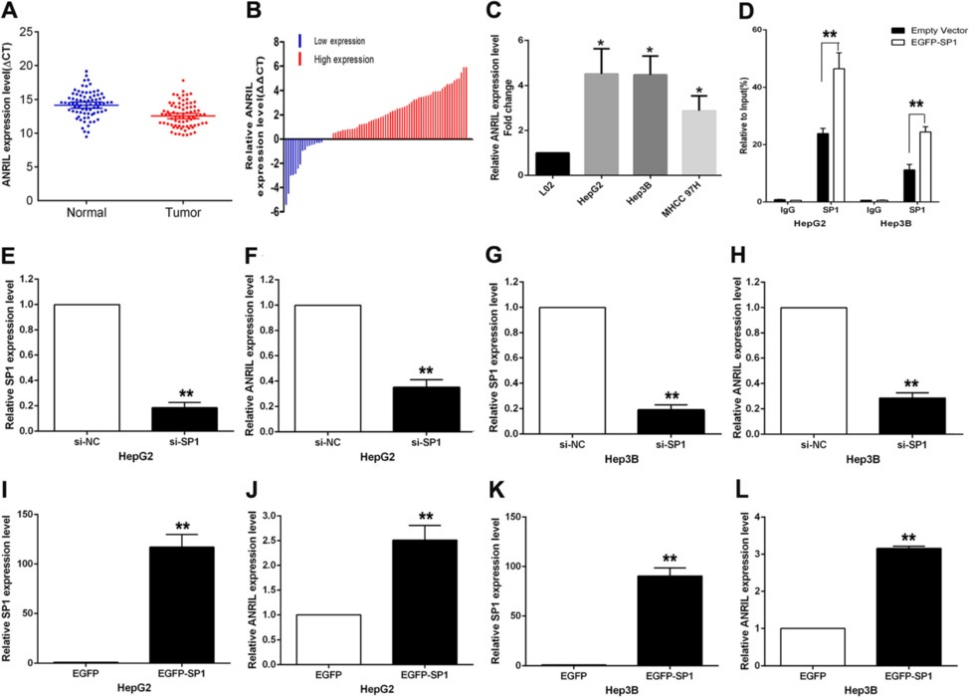
Relative ANRIL expression in HCC tissues and HCC cell lines, and ANRIL regulation by SP1. **(A)** Relative ANRIL expression in HCC tissues (*n* = 77) compared with corresponding non-tumor tissues (*n* = 77). ANRIL expression was examined by qPCR and normalized to GAPDH expression. Results were presented as ΔCT in tumor tissues relative to normal tissues. **(B)** ANRIL expression was classified into two groups. Positive ΔΔCT meant high ANRIL expression. Negative ΔΔCT meant low ANRIL expression. **(C)** Relative ANRIL expression levels of HCC cell lines (HepG2, Hep3B, MHHC-97H) compared with those in the normal hepatic epithelium cell line (L02). **(D)** ChIP-qPCR of SP1 occupancy and binding in the ANRIL promoter in HepG2 and Hep3B cells, and IgG as a negative control. **(E)** The SP1 expression level was determined by qPCR when HepG2 cells were transfected with si-SP1. **(F)** The ANRIL expression level was determined by qPCR when HepG2 cells were transfected with si-SP1. **(G)** The SP1 expression level was determined by qPCR when Hep3B cells were transfected with si-SP1. **(H)** The ANRIL expression level was determined by qPCR when Hep3B cells were transfected with si-SP1. **(I)** The SP1 expression level was determined by qPCR when HepG2 cells were transfected with EGFP-SP1. **(J)** The ANRIL expression level was determined by qPCR when HepG2 cells were transfected with EGFP-SP1. **(K)** The SP1 expression level was determined by qPCR when Hep3B cells were transfected with EGFP-SP1. **(L)** The ANRIL expression level was determined by qPCR when Hep3B cells were transfected with EGFP-SP1. *P < 0.05, **P < 0.01.

ANRIL expression was significantly upregulated in 75.32% (58 of 77, fold ≥1.0) of tumor tissues compared with normal counterparts (*P* < 0.01) (Figure 1A,B). To understand the significance of ANRIL overexpression in HCC, we investigated the potential associations between ANRIL expression and patients’ clinicopathological features. Clinicopathological features of HCC patients are shown in Table 1. Noticeably, high ANRIL expression was significantly correlated with tumor size (*P* < 0.01) and advanced BCLC stage (*P* < 0.01). However, ANRIL expression was not associated with other parameters such as drinking state (*P* = 0.932), age (*P* = 0.850), gender (*P* = 0.608), AFP (*P* = 0.713), HBV (*P* = 0.713), and cirrhosis (*P* = 0.319) in HCC.

**Table 1:** **Correlation between ANRIL expression and clinicopathological characteristics in hepatocellular carcinoma**

**Clinical parameter**	**ANRIL**		**Chi-square test** ***P*** **value**
	**High no. cases**	**Low no. cases**	
Age (years)			0.850
<50	23	8	
>50	35	11	
Gender			0.608
Male	46	14	
Female	12	5	
Drinking state			0.932
Yes	36	12	
No	22	7	
HBV			0.713
Yes	50	17	
No	8	2	
Cirrhosis			0.155
Yes	46	17	
No	12	2	
AFP			0.625
≤20	18	8	
20–400	19	6	
≥400	21	5	
Tumor size			<0.01
≤3 cm	11	9	
3–5 cm	13	9	
5–10 cm	31	1	
≥10 cm	3	0	
BCLC stage			<0.01
0	2	2	
A	19	15	
B	37	2	

### ANRIL is upregulated in HCC cell lines and could be activated by transcript factor SP1

To investigate the functional role of ANRIL in HCC cells, quantitative polymerase chain reaction (qRT-PCR) was used to detect the expression of ANRIL in three HCC cell lines. As shown in Figure 1C, three cell lines (HepG2, HepG3B, MHCC-97H) expressed high levels of ANRIL compared with the normal hepatic epithelium cell line (L02). A previous study indicated that ANRIL expression could be activated by E2F1. In this study, we performed bioinformatics analysis and found that there are 13 SP1 binding sites in the ANRIL promoter region (as shown in Table 2), which suggest that SP1 could also regulate ANRIL transcription in HCC cells. Chromatin immunoprecipitation (ChIP) assay showed that SP1 could directly bind to ANRIL promoter regions (1,081 bp) to silence ANRIL transcription. In addition, overexpression of SP1 in HCC cells could upregulate ANRIL expression, while knockdown of SP1 in HCC cells could downregulate ANRIL expression (as shown in Figure 1D,E,F,G,H,I,J,K).

**Table 2:** **SP1 putative binding sites in the ANRIL promoter region by JASPAR**

**Model ID**	**Model name**	**Score**	**Relative score**	**Start**	**End**	**Strand**	**Predicted site sequence**
MA0079.3	SP1	11.960	0.931611173530848	1241	1251	1	TCTCCTCCTCC
MA0079.3	SP1	11.933	0.931271482620182	1244	1254	1	CCTCCTCCTCC
MA0079.3	SP1	11.615	0.927270678561222	1647	1657	1	GCACCGCCCCC
MA0079.3	SP1	11.673	0.928000384961913	1664	1674	1	TCTCCGCCCCG
MA0079.3	SP1	12.920	0.943689072576764	1702	1712	1	CGCCCGCCCCC
MA0079.3	SP1	10.466	0.912814943140641	1709	1719	1	CCCCCACCTTC
MA0079.3	SP1	14.400	0.962309166939219	1721	1731	1	CCCCCACCCCC
MA0079.3	SP1	11.514	0.925999982932433	1727	1737	1	CCCCCACCCCA
MA0079.3	SP1	13.360	0.949224776306143	1732	1742	1	ACCCCACCCCC
MA0079.3	SP1	10.179	0.909204154571706	1864	1874	1	CTCCCGCCTAC
MA0079.3	SP1	9.496	0.90061123264633	1882	1892	1	TTCCCGCCCTG
MA0079.3	SP1	14.400	0.962309166939219	1899	1909	1	CCCCCACCCCC
MA0079.3	SP1	10.467	0.912827524285481	1919	1929	1	TTCCCACCCTC

Thirteen putative sites were predicted with these settings (90%) in sequence named gi 568815589: 21992791–21994791. Comment: This type of analysis has a high sensitivity but abysmal selectivity. In other words, while true function will be detected in most cases, most predictions will correspond to sites bound *in vitro* but with no function *in vivo*. A number of additional constraints of the analysis can improve the prediction; phylogenetic footprinting is the most common. We recommend using the ConSite service, which uses the JASPAR datasets. The review Nat Rev Genet. 2004 Apr;5(4):276–87 gives a comprehensive overview of transcription binding site prediction [34].

### Knockdown of ANRIL inhibits HCC cell proliferation and induces cell apoptosis *in vitro*

**Figure 2:**
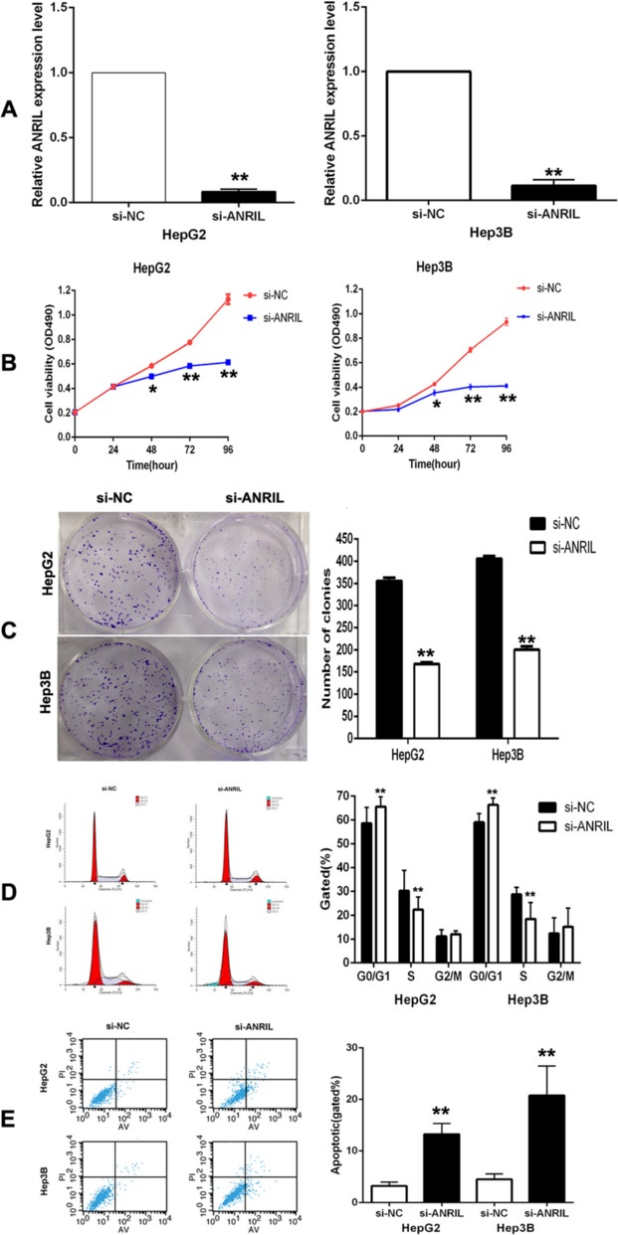
Effects of knockdown of ANRIL on HCC cell viability and apoptosis *in vitro*. **(A)** The ANRIL expression level was determined by qPCR when HepG2 and Hep3B cells were transfected with si-ANRIL. **(B)** MTT assays were used to determine the cell viability for si-ANRIL-transfected HepG2 and Hep3B cells. Values represented the mean ± s.d. from three independent experiments. **(C)** Colony formation assays were conducted to determine the proliferation of si-ANRIL-transfected HepG2 and Hep3B cells. **(D)** Flow cytometry assays were performed to analyze the cell cycle progression when HCC cells were transfected with si-ANRIL 24 h later. The bar chart represented the percentage of cells in G0/G1, S, or G2/M phase, as indicated. **(E)** Flow cytometry assays were performed to analyze the cell apoptosis when HCC cells were transfected with si-ANRIL 48 h later. *P < 0.05, **P < 0.01.

To investigate the potential role of ANRIL on HCC cell proliferation, ANRIL siRNA was transfected into HepG2 and HepG3B cells. To ensure the efficiency of interference and avoid off-target effects, we used a validated effective interference target sequence of ANRIL, according to Kotake’s study [12]. qRT-PCR assays revealed that ANRIL expression was significantly reduced after transfection with si-ANRIL (Figure 2A). Then, MTT assay showed that knockdown of ANRIL expression significantly inhibited cell proliferation both in HepG2 and HepG3B cells compared with control cells (Figure 2B). Similarly, the result of colony formation assay revealed that clonogenic survival was significantly decreased following inhibition of ANRIL both in HepG2 and Hep3B cell lines (Figure 2C). Next, flow cytometry analysis was performed to further examine the effect of ANRIL on proliferation of HCC cells by altering cell cycle progression or apoptosis. The results revealed that the cell cycle progression of HepG2/si-ANRIL and Hep3B/si-ANRIL cells was significantly stalled at the G1-G0 phase compared with cells transfected with si-NC (Figure 2D). In addition, knockdown of ANRIL could obviously induce cell apoptosis (Figure 2E).

### Effect of ANRIL on HCC cell migration and invasion

**Figure 3:**
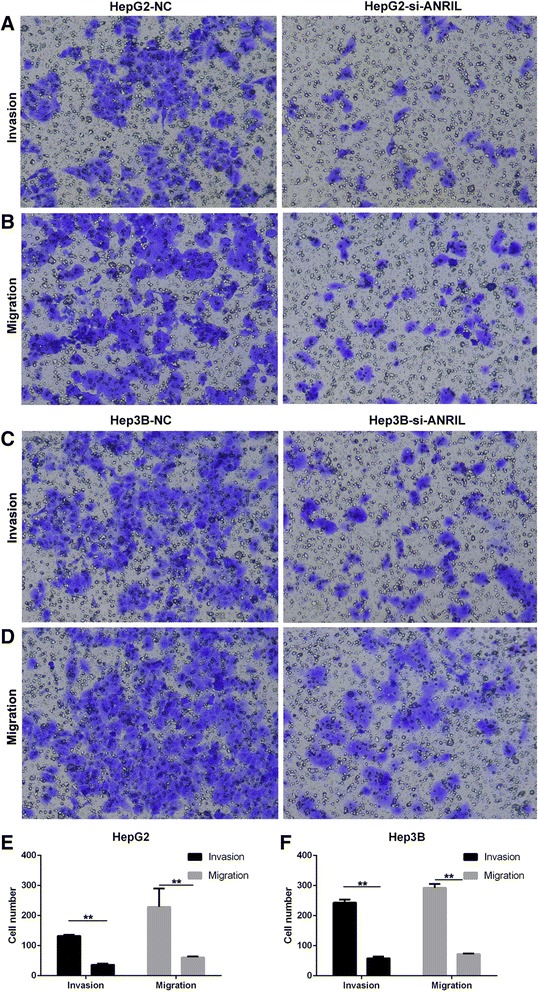
Effect of ANRIL on HCC cell migration and invasion. **(A,B,E)** The results showed that inhibition of ANRIL could significantly impair HepG2 cell migration and invasion ability when compared with control cells. **(C,D,F)** The results showed that inhibition of ANRIL could significantly impair Hep3B cell migration and invasion ability when compared with control cells. **P < 0.01.

Migration and invasion are significant aspects of cancer progression, which involve the dissolution of extracellular matrix proteins and the migration of tumor cells into contiguous tissues. To investigate whether ANRIL had a direct functional role in cell invasion in HCC, we performed transwell assays. The results showed that inhibition of ANRIL could significantly impair HCC cell migration and invasion ability when compared with control cells (Figure 3).

### ANRIL promotes HCC cell proliferation *in vivo*

**Figure 4:**
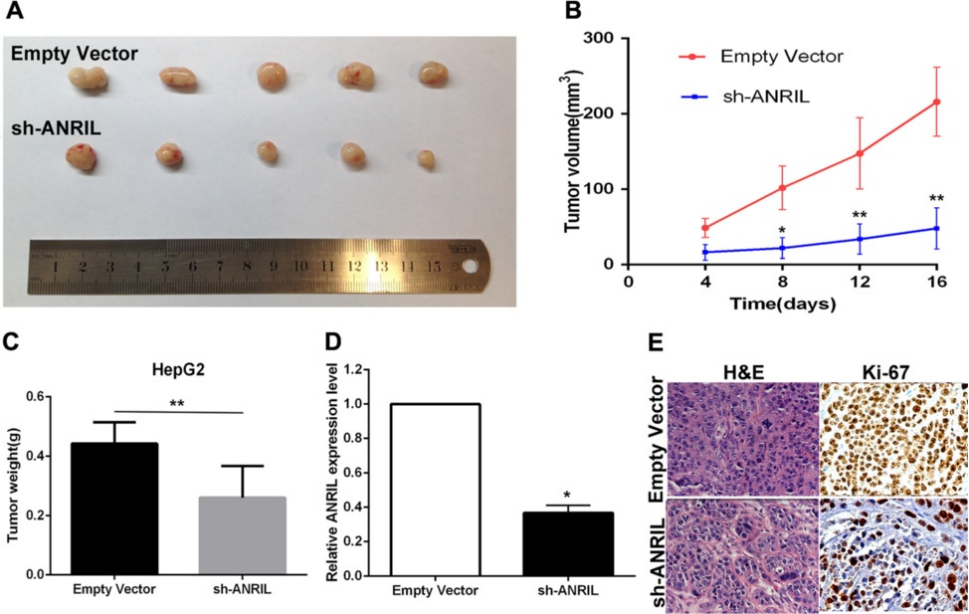
Effects of downregulation of ANRIL on tumor growth *in vivo*. **(A)** Tumors from mice 16 days after injection of HepG2 cells stably transfected with sh-ANRIL or empty vector. **(B)** The tumor volume was calculated every 4 days after injection of HepG2 cells stably transfected with sh-ANRIL or empty vector. Points, mean (*n* = 5); bars indicate s.d. **(C)** Tumor weights are represented as means of tumor weights ± s.d. **(D)** qPCR analysis of ANRIL expression in tumor tissues formed from HepG2/sh-ANRIL and HepG2/empty vector. **(E)**. Tumors developed from sh-ANRIL-transfected HepG2 cells showed lower Ki-67 protein levels than tumors developed by control cells. Left: H&E staining. Right: immunostaining. *P < 0.05, **P < 0.01.

To further determine whether ANRIL affects tumorigenesis, we injected HepG2 cells transfected with either empty vector or sh-ANRIL into male nude mice. Consistent with *in vitro* results, tumor growth in the sh-ANRIL group was obviously slower than that in the empty vector group (Figure 4A). Up to 16 days after injection, the average tumor weight in the sh-ANRIL group (0.260 ± 0.107 g) was significantly lower than that in the control group (0.442 ± 0.716 g) (*P* < 0.01) (Figure 4B). qRT-PCR analysis was performed to detect the average expression of ANRIL in tumor tissues selected from mice (Figure 4C). Results demonstrated that the average expression levels of ANRIL in the sh-ANRIL group were lower than those in the empty group. Moreover, we found that the tumors developed from empty vector-transfected cells showed a stronger Ki-67 expression than tumors formed from sh-ANRIL as detected by immunohistochemistry (IHC) analysis (Figure 4D). These data further supported the role of ANRIL in HCC cell growth and proliferation.

### ANRIL negatively regulates expression of KLF2

**Figure 5:**
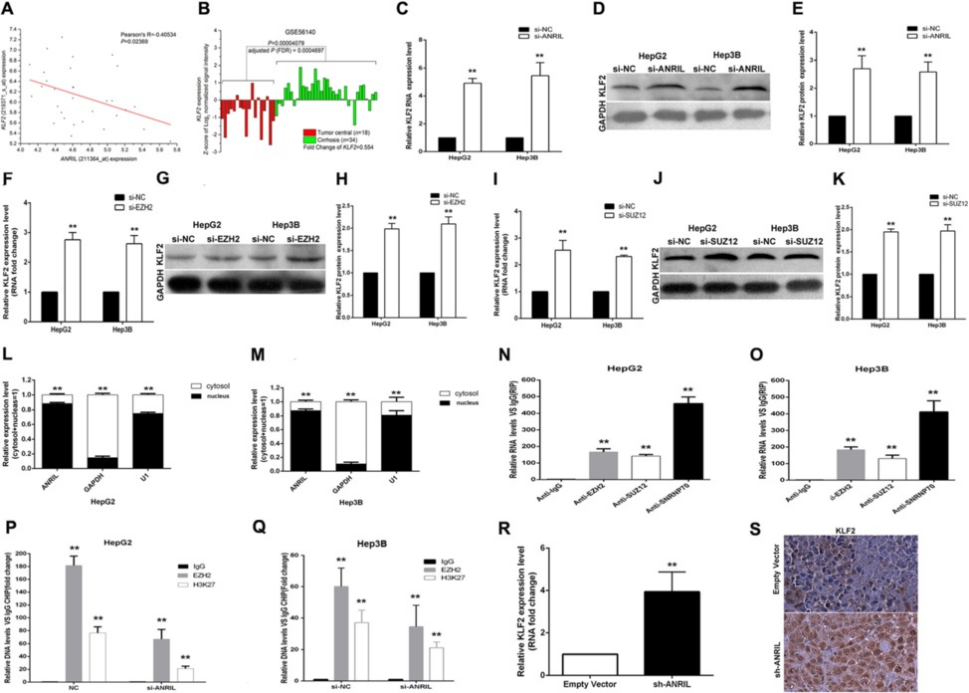
ANRIL could silence KLF2 expression. **(A)** Co-expression analysis by using GSE45435 data from GEO datasets. **(B)** The KLF2 gene expression in HCC by using GSE 56140. **(C-E)** The levels of KLF2 mRNA and protein were detected by qPCR and Western blot when HepG2 and Hep3B cells were transfected with si-ANRIL, and results are expressed relative to the corresponding values for control cells. **(F-H)** The levels of KLF2 mRNA and protein were detected by qPCR and Western blot when HepG2 and Hep3B cells were transfected with si-EZH2, and results are expressed relative to the corresponding values for control cells. **(I-K)** The levels of KLF2 mRNA and protein were detected by qPCR and Western blot when HepG2 and Hep3B cells were transfected with si-SUZ12, and results are expressed relative to the corresponding values for control cells. **(L,M)** ANRIL expression levels in cell cytoplasm or nucleus of HCC cell lines Hep3B and HepG2 were detected by qPCR. **(N,O)** RIP with rabbit monoclonal anti-EZH2, anti-SUZ12, anti-SNRNP70, and preimmune IgG from HepG2 and Hep3B cell extracts. RNA levels in immunoprecipitates were determined by qPCR. Expression levels of ANRIL RNA were presented as fold enrichment in EZH2 and SUZ12 relative to IgG immunoprecipitates; relative RNA levels of U1 snRNA in SNRNP70 relative to IgG immunoprecipitates were used as positive control. **(P,Q)** ChIP-qPCR of EZH2 occupancy and H3K27-3me binding in the KLF2 promoter in HepG2 cells, and IgG as a negative control; ChIP-qPCR of EZH2 occupancy and H3K27-3me binding in the KLF2 promoter in HepG2 cells transfected with ANRIL siRNA (48 h) or scrambled siRNA. **(R)** The KLF2 expression level was determined by qPCR in mice tumors formed from HepG2/sh-ANRIL and HepG2/empty vector. **(S)** Tumors developed from sh-ANRIL-transfected HepG2 cells showed higher KLF2 protein levels than tumors developed by control cells. **P < 0.01.

As previously reported, ANRIL could suppress p15 and p21 expression by binding with PRC2. In the present study, to investigate whether there are some other target genes that may be regulated by ANRIL, we performed co-expression analysis by using GSE45435 data from GEO datasets. The results showed that KLF2 may be a new target of ANRIL in HCC (as shown in Figure 5A). We also analyzed the KLF2 gene expression in HCC by using GSE 56140. It showed that KLF2 was downregulated in HCC (as shown in Figure 5B). And we further found that knockdown of ANRIL expression could upregulate both KLF2 mRNA and protein expression levels in HCC cells (Figure 5C, D, E). Moreover, knockdown of EZH2 or SUZ12 could also upregulate KLF2 mRNA and protein expression levels in HCC cells (Figure 5F,G,H,I,J,K). We examined the ANRIL expression levels in the HCC cell cytoplasm and nucleus distribution, and the results showed that ANRIL expression is more located in the nucleus (seen in Figure 5L,M). In addition, the results of RNA immunoprecipitation (RIP) assays revealed that ANRIL could directly bind with PRC2 in HCC cells (seen in Figure 5N,O). And ChIP assays were performed to determine whether EZH2 could directly bind to KLF2 promoter regions to silence KLF2 transcription. The results showed that EZH2 can directly bind to KLF2 promoter regions (616 bp), while knockdown of ANRIL expression decreased its binding ability (seen in Figure 5P,Q). Then, qRT-PCR analysis was performed to detect the average expression of KLF2 in tumor tissues from mice (Figure 5R). Results demonstrated that the average expression levels of KLF2 in the sh-ANRIL group were higher than those in the empty group. Finally, we found that the tumors developed from sh-ANRIL-transfected cells showed a stronger KLF2 expression than tumors formed from the empty vector as detected by IHC analysis (Figure 5S). These data indicated that KLF2 was a new ANRIL target gene in HCC, and its expression can be silenced by EZH2 which is recruited by ANRIL to the KLF2 promoter region and mediated H3K27 trimethylation modification.

### Overexpression of KLF2 impairs HCC cell proliferation and induces cell apoptosis

**Figure 6:**
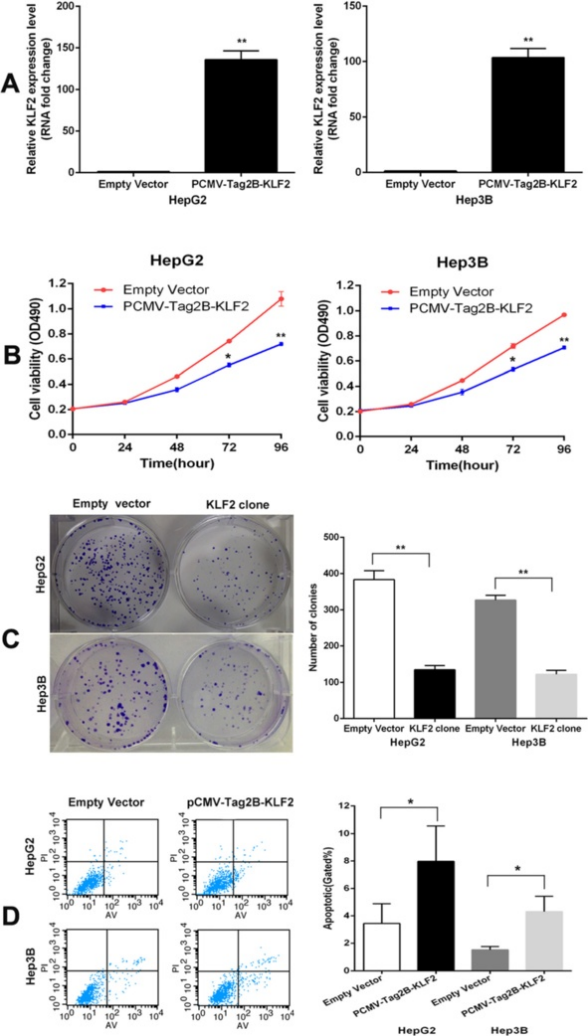
Overexpression of KLF2 expression inhibits HepG2 cell proliferation and improves apoptosis. **(A)** The mRNA level of KLF2 in HepG2 and Hep3B cells transfected with pCMV-Tag2B-KLF2 or empty vector was detected by qPCR. **(B,C)** MTT assays and colony formation assays were used to determine the cell viability for pCMV-Tag2B-KLF2-transfected or empty vector-transfected HepG2 and Hep3B cells. Values represent the mean ± s.d. from three independent experiments. **(D)** Apoptosis was determined by flow cytometry. UL, necrotic cells; UR, terminal apoptotic cells; LR, early apoptotic cells. *P < 0.05 and **P < 0.01.

To determine whether KLF2 is involved in ANRIL-mediated increase in HCC cell proliferation, we upregulated KLF2 expression in HCC cells by transfecting with a FLAG-tagged KLF2 expression vector using the pCMV-Tag2B vector (Stratagene, Santa Clara, CA, USA). The qRT-PCR results showed that KLF2 expression is significantly upregulated in pCMV-Tag2B-KLF2-transfected HCC cells when compared with control cells (Figure 6A). Furthermore, MTT assays and colony formation assay revealed that KLF2 overexpression inhibited HCC cell growth (Figure 6B,C), and flow cytometry analysis indicated that increased KLF2 expression induced cell apoptosis. These data suggest that KLF2 was partly involved in HCC cell proliferation and apoptosis.

### ANRIL negatively regulates expression of KLF2 by rescue assays

**Figure 7:**
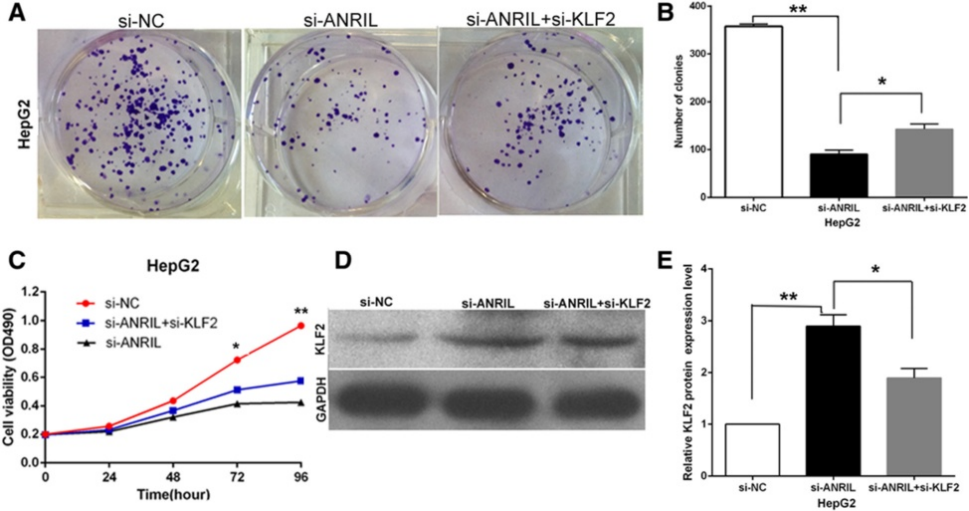
ANRIL negatively regulates expression of KLF2 by rescue assays. **(A,B)** Colony formation assays were used to determine the cell viability for HepG2 cells transfected with si-NC and si-ANRIL and co-transfected with siANRIL and si-KLF2. Values represent the mean ± s.d. from three independent experiments. **(C)** MTT assays were used to determine the cell viability for HepG2 cells transfected with si-NC and si-ANRIL and co-transfected with siANRIL and si-KLF2. Values represent the mean ± s.d. from three independent experiments. **(D,E)** The levels of KLF2 protein levels were determined by Western blot when HepG2 cells were transfected with si-NC and si-ANRIL and co-transfected with siANRIL and si-KLF2.*P < 0.05, **P < 0.01.

Rescue assays were performed to determine whether ANRIL regulates HCC cell proliferation via repressing KLF2 expression. HepG2 cells were co-transfected with si-ANRIL and si-KLF2. The results of MTT and colony formation assays indicated that co-transfection could partially rescue si-ANRIL-impaired proliferation in HepG2 cells (Figure 7A,B). Western blot showed the same results (Figure 7C).

## Discussion

In recent years, the discovery of lncRNAs, which have emerged as a new and crucial layer of gene regulators, has dramatically altered our understanding of the biology of complex diseases including cancers [16,17]. A large number of studies have shown that dysregulated expression of lncRNAs participates in cancer progression and predicts patients’ outcome [18-20]. For example, GAS5 can promote the apoptosis of prostate cancer cells, and its levels decline as prostate cancer cells acquire castrate resistance, so that enhancing GAS5 expression may improve the effectiveness of chemotherapies [6]. In HCC, HULC was the first reported lncRNA that is specifically upregulated [21,22]. A number of lncRNAs, such as MVIH and URHC, have been reported to be involved in HCC development and progression [23,24]. In this study, we found another lncRNA, ANRIL, whose expression is significantly upregulated in HCC tissues compared with normal tissues. Moreover, increased ANRIL expression was correlated with HCC tumor size and BCLC stage, which suggests that ANRIL may play a key role in HCC development and progression.

Recently, several studies indicated that lncRNA expression could also be regulated by some transcript factors (TF); for example, c-myc could activate HOTAIR transcription, and PVT-1 expression can be regulated by p53 [25,26]. ANRIL expression has been reported to be regulated by a key TF, E2F1 [13,27]; however, in this study, we performed bioinformatics analysis and found that SP1 could also regulate ANRIL transcription in HCC cells. The ChIP assay also showed that SP1 could directly bind to ANRIL promoter regions to silence ANRIL transcription. In addition, overexpression of SP1 in HCC cells could upregulate ANRIL expression, while knockdown of SP1 in HCC cells could downregulate ANRIL expression. These data showed that ANRIL expression could also be regulated by SP1 in HCC cells, which suggests that one lncRNA may be simultaneously regulated by multiple different transcript factors.

As is known, lncRNAs participated in cancer cells’ biological function, and we found that knockdown of ANRIL could impair HCC cell proliferation and invasion and induce cell apoptosis both *in vitro* and *in vivo*. These data suggest that lncRNA ANRIL contributes to HCC development via regulation of cell proliferation and apoptosis. A completely different mode of action is executed by the lncRNA ANRIL to block the activity of tumor suppressor genes. For example, ANRIL interacts with SUZ12 (a subunit of the PRC2) and recruits the complex to repress the expression of p15 (INK4B), a well-known tumor suppressor gene [13]. A similar study identified CBX7 (chromobox homolog 7), a subunit of the polycomb repressive complex 1 (PRC1), as a molecular interaction partner of ANRIL, which results in the recruitment of PRC1 to the p16(INK4A)/p14(ARF) locus and silencing of this gene locus by H3K27 trimethylation [10]. However, we found that ANRIL could bind with both EZH2 and SUZ12 in HCC cells. Furthermore, bioinformatics analysis indicated that KLF2 could be a new ANRIL downstream target, and knockdown of ANRIL and EZH2 and SUZ12 expression indeed both upregulated KLF2 expression levels in HCC cells. In addition, ChIP assays also demonstrated that EZH2 could directly bind to the KLF2 promoter region and inhibition of ANRIL decreased its binding ability. Our results indicated that ANRIL could repress KLF2 transcription by binding with EZH2 and SUZ12 and recruitment of PRC2 to the KLF2 gene locus in HCC cells.

The Kruppel-like factor (KLF) family which consists of a set of transcription factors that have been identified in diverse organisms functions in cell differentiation and proliferation [28]. They have been identified as suppressors or activators of different genes in a cell type and promoter-dependent manner [29]. KLF2 is one of the critical members due to its tumor suppressor function in tumors [30,31]. Moreover, a previous study showed that EZH2 could directly bind to the KLF2 promoter and silence of KLF2 expression results in blocking the tumor suppressor features of KLF2, which is partly mediated by p21 [32]. Our data also showed that ANRIL could take part in HCC cell proliferation by silencing KLF2 transcription, and KLF2 overexpression further led to the decreased HCC cell proliferation and increased cell apoptosis. Furthermore, we performed rescue assays to determine whether ANRIL regulates HCC cell proliferation via repressing KLF2 expression. The results of MTT and colony formation assays indicated that co-transfection could partially rescue si-ANRIL-impaired proliferation in HepG2 cells. These data indicate that ANRIL promotes HCC cell proliferation through the downregulation of KLF2 expression. Our results suggested that lncRNA, especially ANRIL, may influence the same cell biological function via regulating different target genes depending on different cancer cells.

## Conclusion

In summary, the expression of ANRIL was significantly upregulated in HCC tissues and cells, suggesting that its overexpression may be an important factor for HCC progression. We showed that ANRIL may regulate the proliferation ability of HCC cells partially through silencing of KLF2 by binding with PRC2, which suggested that lncRNAs contribute to different cancer cells’ biological function through regulating different genes. Further insights into the functional and clinical implications of ANRIL and its targets, which are identified as KLF2, may contribute to the understanding of HCC pathogenesis and facilitate the development of lncRNA-directed diagnostics and therapeutics against this disease.

## Materials and methods

### Patient data and tissue samples

A total of 77 fresh HCC tissue samples and matched normal adjacent tissue samples were selected from patients who underwent resection of HCC at Huai’an First People’s Hospital, Nanjing Medical University (Huai’an, China). The HCC diagnosis was histopathologically confirmed. None of the patients received preoperative therapy. Data from all subjects were obtained from medical records, pathology reports, and personal interviews with the subjects. The collected data included gender, age, drinking state, the history of HBV and cirrhosis, and HCC features (e.g., tumor size, stage). HCC clinical stage was determined according to the BCLC staging classification based on the article by Bruix and Llovet [33]. The clinical information for all of the samples is detailed in Table 1. Fresh samples were snap-frozen in liquid nitrogen immediately after resection and stored at −80°C. Matched non-tumor specimens were obtained from a part of the resected specimen that was farthest from the tumor.

### Ethical approval of the study protocol

This study was conducted according to the principles expressed in the Declaration of Helsinki. Tissue specimen collections were made with full informed consent of all patients following institutional ethical guidelines that were reviewed and approved by Huai’an First People’s Hospital, Nanjing Medical University (Huai’an, China).

### Cell culture

Human HCC cell lines (HepG2, Hep3B, MHCC-97H) and one normal hepatic epithelial cell line (L02, control) were provided by Dr. Beicheng Sun from the Department of Hepatopancreatobiliary, First Affiliated Hospital, Nanjing Medical University (Nanjing City, Jiangsu Province, People’s Republic of China). All cell lines were cultured in DMEM (GIBCO-BRL) medium supplemented with 10% fetal bovine serum (FBS) at 37°C in 5% CO_2_.

### RNA extraction and qRT-PCR analysis

The total RNA was extracted from tissues or cells with TRIzol reagent (Invitrogen, Grand Island, NY, USA), according to the manufacturer’s protocol. One microgram total RNA was reverse transcribed in a final volume of 20 μL under standard conditions using PrimeScript RT Reagent Kit with gDNA Eraser (Takara, Dalian, China; RR047A). After the RT reaction, 1 μL of the complementary DNA was used for subsequent qRT-PCR reactions (SYBR Premix Ex Taq, TaKaRa) following the manufacturer’s protocol. The results were normalized to the expression of GAPDH. The qRT-PCR and data collection were carried out on an ABI 7500 real-time PCR system (Applied Biosystems, Foster City, CA, USA), and results were analyzed and expressed relative to threshold cycle (CT) values and then converted to fold changes. All primer sequences are summarized in Additional file 1: Table S1.

### Transfection of cell lines

HCC cell lines were transfected with specific siRNA oligonucleotides, ANRIL siRNA, and to avoid off-target effects and ensure the efficiency of interference, we used an indeed effective interference target sequence of ANRIL, according to a previous study [12]. EZH2 siRNA and SUZ12 siRNA were purchased from Realgene (Nanjing, China). Non-specific siRNA (si-NC) and si-ANRIL were purchased from Invitrogen. Typically, cells were seeded in six-well plates and then transfected the next day with specific siRNA (100 nM) and control siRNA (100 nM) by using Lipofectamine RNAi MAX, according to the manufacturer’s protocol (Invitrogen). EGFP-SP1 was purchased from Add gene. Plasmid vectors (EGFP-SP1, sh-ANRIL pCMV-Tag2B-FLAG-KLF2, and empty vector) for transfection were prepared using DNA Midiprep or Midiprep kits (Qiagen, Hilden, Germany) and transfected into HepG2 and Hep3B cells.

### Cell proliferation assays

Cell proliferation was monitored using Cell Proliferation Reagent Kit I (MTT) (Roche, Basel, Switzerland). The transfected cells were plated in 96-well plates (3,000 cells/well). Cell proliferation was determined every 24 h following the manufacturer’s protocol. For the colony formation assay, 500 transfected cells were placed into each well of a six-well plate and maintained in DMEM containing 10% FBS for 12 days, replacing the medium every 4 days. Colonies were fixed with methanol and stained with 0.1% crystal violet (Sigma-Aldrich, St. Louis, MO, USA) in PBS for 15 min. The colony formation was determined by counting the number of stained colonies. Triplicate wells were measured in each treatment group.

### Flow cytometry for cell cycle analysis

HepG2 or Hep3B cells for cell cycle analysis were collected 24 h after being transfected with si-ANRIL or respective control, and 48 h after being transfected with pCMV-Tag2B-KLF2 or empty vector. Then, cells were stained with propidium iodide (PI) using the CycleTEST™ PLUS DNA Reagent Kit (BD Biosciences) following the protocol and analyzed by FACScan. The percentage of the cells in G0/G1, S, and G2/M phases was counted and compared.

### Flow cytometry for cell apoptosis analysis

HepG2 or Hep3B cells transfected with si-ANRIL, pCMV-Tag2B-KLF2, or respective control were harvested 48 h and then collected. After double staining with FITC-Annexin V and PI was done using the FITC Annexin V Apoptosis Detection Kit (BD Biosciences) according to the manufacturer’s protocol, the cells were analyzed with a flow cytometry system (FACScan®, BD Biosciences) equipped with CellQuest software (BD Biosciences). Cells were discriminated into viable cells, dead cells, early apoptotic cells, and apoptotic cells, and then the relative ratio of early apoptotic cells were compared to control transfectant from each experiment.

### Cell migration and invasion assays

HepG2 or Hep3B cells transfected with si-ANRIL or respective control were harvested 48 h and then collected. For the migration assays, 5 × 10^4^ cells in serum-free medium were placed into the upper chamber of an insert (8-μm pore size, Millipore). For the invasion assays, 1 × 10^5^ cells in serum-free medium were placed into the upper chamber of an insert coated with Matrigel (Sigma-Aldrich). A medium containing 10% FBS was added to the lower chamber. After incubation for 24 h, we removed the cells remaining on the upper membrane with cotton wool. Cells that had migrated to or invaded the membrane were fixed with methanol, stained with 0.1% crystal violet, and imaged and counted using an IX71 inverted microscope (Olympus, Tokyo, Japan). Experiments were repeated three times.

### Xenograft study

HepG2 cells were transfected with sh-ANRIL or Scramble using Lipofectamine 2000 (Invitrogen). Forty-eight hours later, cells were collected and injected into either side of the posterior flank of the male BALB/c nude mice (4–5 weeks old). Mice were purchased from Shanghai Experimental Animal Center of the Chinese Academy of Sciences. The tumor volumes and weights were measured every 4 days in mice from the control (five mice) or sh-ANRIL (five mice) groups, and tumor volumes were calculated by using the equation *V* = 0.5 × *D* × *d* ^2^ (*V*, volume; *D*, longitudinal diameter; *d*, latitudinal diameter). Sixteen days after injection, the mice were killed, and tumors were collected for further study (weight measure, RNA extraction, and IHC). This study was carried out strictly in accordance with the recommendations in the Guide for the Care and Use of Laboratory Animals of the National Institutes of Health. The protocol was approved by the Committee on the Ethics of Animal Experiments of Nanjing Medical University.

### Immunohistochemistry

Tumors from mice were immunostained for HE, Ki-67, and KLF2. The signal was amplified and visualized with 3′-diaminobenzidine chromogen, followed by counterstaining with hematoxylin. Expression was considered to be positive when 50% or more tumor cells were stained. Anti-Ki-67 (1:50) and anti-KLF2 (1:50) were purchased from R & D company.

### Western blot assay

Cells were lysed by using mammalian protein extraction reagent RIPA (Beyotime, Haimen, China) supplemented with protease inhibitor cocktail (Roche). Fifty micrograms of the protein extractions were separated by 10% SDS-PAGE transferred to 0.22-mm nitrocellulose (NC) membranes (Sigma-Aldrich) and incubated with specific antibodies. The autoradiograms were quantified by densitometry (Quantity One software, Bio-Rad, Hercules, CA, USA). Anti-KLF2 was purchased from Sigma (1:1,000). Results were normalized to the expression GAPDH (mouse anti-GAPDH) (Sigma; 1:1,000).

### Subcellular fractionation location

The separation of the nuclear and cytosolic fractions of HCC cell lines was performed according to the protocol of the PARIS Kit (Life Technologies, Carlsbad, CA, USA).

### Chromatin immunoprecipitation assays

The ChIP assays were performed by using the EZ-ChIP Kit according to the manufacturer’s instruction (Millipore, Billerica, MA, USA). HepG2 and Hep3B cells were treated with formaldehyde and incubated for 10 min to generate DNA-protein cross-links. Cell lysates were then sonicated to generate chromatin fragments of 200–300 bp and immunoprecipitated with EZH2-, SUZ12-, and H3K27me3-specific antibody (CST) or IgG as control. Precipitated chromatin DNA was recovered and analyzed by qRT-PCR.

### RNA immunoprecipitation

RIP experiments were performed by using a Magna RIP RNA-Binding Protein Immunoprecipitation Kit (Millipore) according to the protocol. Antibody for RIP assays of EZH2 and SUZ12 was purchased from Millipore.

### Statistical analysis

All statistical analyses were performed by using SPSS 17.0 software (IBM, Chicago, IL, USA). The significance of differences between groups was estimated by the Student *t*-test, Wilcoxon test, or *χ* ^2^ test. Two-sided *P* values were calculated, and differences were considered to be statistically significant at *P* < 0.05. Kendall’s Tau-b and Pearson correlation analyses were used to investigate the correlation between ANRIL and KLF2 expressions.

## Additional file

Additional file 1: Table S1. Sequence of primers and siRNA.

## Abbreviations

lncRNA: Long non-coding RNA
ANRIL: CDKN2B antisense RNA1
HCC: Hepatocellular carcinoma
BCLC: Barcelona Clinic Liver Cancer
PCR: Polymerase chain reaction
RIP: RNA immunoprecipitation
ChIP: Chromatin immunoprecipitation
GAPDH: Glyceraldehyde-3-phosphate dehydrogenase
KLF2: Kruppel-like factor 2
